# Correction to: Cardiovascular risk factors and body composition in adults with achondroplasia

**DOI:** 10.1038/s41436-021-01201-1

**Published:** 2021-07-07

**Authors:** Svein O. Fredwall, Jennifer Linge, Olof Dahlqvist Leinhard, Lisa Kjønigsen, Heidi Beate Eggesbø, Harald Weedon-Fekjær, Ingeborg Beate Lidal, Grethe Månum, Ravi Savarirayan, Serena Tonstad

**Affiliations:** 1 Sunnaas Rehabilitation Hospital, TRS National Resource Centre for Rare Disorders, Nesodden, Norway; 2 Faculty of Medicine, Institute of Clinical Medicine, University of Oslo, Oslo, Norway; 3 AMRA Medical AB, Linköping, Sweden; 4 Department of Health, Medicine and Caring Sciences, University of Linköping, Linköping, Sweden; 5 Center for Medical Image Science and Visualization, University of Linköping, Linköping, Sweden; 6 Oslo University Hospital, Division of Radiology and Nuclear Medicine, Oslo, Norway; 7 Oslo Centre for Biostatistics and Epidemiology, Research Support Service, Oslo University Hospital, Oslo, Norway; 8 Department of Research, Sunnaas Rehabilitation Hospital, Nesodden, Norway; 9 Murdoch Children’s Research Institute and University of Melbourne, Parkville, Australia; 10 Department of Preventive Cardiology, Oslo University Hospital, Oslo, Norway

Correction to: *Genetics in Medicine* (2021) **23**:732–739; 10.1038/s41436-020-01024-6; Article published online 18 November 2020

The article “Cardiovascular risk factors and body composition in adults with achondroplasia”, written by Svein O. Fredwall, Jennifer Linge, Olof Dahlqvist Leinhard, Lisa Kjønigsen, Heidi Beate Eggesbø, Harald Weedon-Fekjær, Ingeborg Beate Lidal, Grethe Månum, Ravi Savarirayan, and Serena Tonstad, was originally published Online First without Open Access. After publication in volume 23, issue 4, page 732–739 the author decided to opt for Open Choice and to make the article an Open Access publication. Therefore, the copyright of the article has been changed to © The Author(s) 2021 and the article is forthwith distributed under the terms of the

Creative Commons Attribution-NonCommercial-NoDerivatives 4.0 International License, which permits any non-commercial use, sharing, distribution and reproduction in any medium or format, as long as you give appropriate credit to the original author(s) and the source, and provide a link to the Creative Commons licence. You do not have permission under this licence to share adapted material derived from this article or parts of it.

The images or other third party material in this article are included in the article’s Creative Commons licence, unless indicated otherwise in a credit line to the material. If material is not included in the article’s Creative Commons licence and your intended use is not permitted by statutory regulation or exceeds the permitted use, you will need to obtain permission directly from the copyright holder.

To view a copy of this licence, visit http://creativecommons.org/licenses/by-nc-nd/4.0/.

